# Lead Free Multilayered Polymer Composites for Radiation Shielding

**DOI:** 10.3390/polym14091696

**Published:** 2022-04-21

**Authors:** Laurynas Gilys, Egidijus Griškonis, Paulius Griškevičius, Diana Adlienė

**Affiliations:** 1 Department of Physics, Faculty of Mathematics and Natural Sciences, Kaunas University of Technology, Studentu Street 50, LT-51368 Kaunas, Lithuania; diana.adliene@ktu.lt; 2 Department of Physical and Inorganic Chemistry, Faculty of Chemical Technology, Kaunas University of Technology, Radvilenu Street 19, LT-50254 Kaunas, Lithuania; egidijus.griskonis@ktu.lt; 3 Department of Mechanical Engineering, Faculty of Mechanical Engineering and Design, Kaunas University of Technology, Studentu Street 56, LT-51424 Kaunas, Lithuania; paulius.griskevicius@ktu.lt

**Keywords:** lead-free, multi-layered structure, polymer composites, X-ray attenuation, radiation shielding and radiation protection

## Abstract

Silicone-based polymer composites containing high atomic number additives are prioritized for the development of new materials for radiation shielding, due to their mechanical, thermal, electrical, and multifunctional properties. The X-ray attenuation properties, as well as mechanical properties, of the newly developed-lead-free multi-layered structures for radiation shielding, based on silicone composite layers containing tin, cerium oxide, tungsten oxide, and bismuth additives, are analyzed and discussed in this paper. It is shown that, by varying the additive concentrations in silicone composites, lead-free and flexible layered structures, exhibiting lead-equivalent X-ray shielding, can be fabricated.

## 1. Introduction

The rapid development of medical radiation technologies and the growing number of radiation diagnostics procedures has increased the concern about the adequate radiation protection of patients and personnel, using appropriate shielding equipment. In general, leaded rubber aprons are the most common shielding equipment that are used for the radiation protection of personnel performing low energy (40–150 keV) diagnostic and interventional radiology procedures. Despite its great ionizing radiation shielding properties, lead is highly toxic and may cause harmful health effects; it requires a high-cost recycling procedure; and leaded aprons and other leaded protective garment are heavy to wear and may cause significant health problems. On the other hand, intensive daily use and sometimes a lack of careful handling of protective garments may result in structural damages of a specific leaded rubber material; thus, causing reduced radiation protection effectiveness [1,2].

Polymer composites containing high Z additives, such as bismuth, tungsten, tantalum, and other materials, are promising candidates for lead replacement in protective garments, since these materials are characterized by good X-ray absorption, mechanical, and physical properties [3].

The selection of composite fillers and polymers enables the formation of mechanically stable, but also flexible, solid materials and allows for modification of the chemical composition and physical properties of polymeric composites using easy-to-control fabrication parameters. Polyethylene, ultra-high molecular weight polyethylene, polylactic acid, epoxy, raw rubber and silicon rubber, polydimethylsiloxane and many other polymers are commonly used as a matrix for fabrication of polymeric composites for radiation shielding [4,5,6,7,8,9]. The development of lead-free, lightweight, safe, robust, and reliable radiation shielding composites [10,11,12,13], as well as the development of new technologies for their fabrication, has made tremendous progress in recent decades [14,15].

Different polymer modifications have been made over the past century, in order to increase resistance to radiation, including radiation cross-linking, radiation-induced polymerization, and polymer degradation. Likewise, medical products to be sterilized by radiation are often made from polymeric materials, which must be resistant to the administered dose. According to the research done in the past, such polymers as fluoro-rubber, silicone rubber, ethylene-propylene rubber, and urethane rubber are able to resist radiation doses greater than 500 kGy. At higher doses, decreased elongation and increased hardness of the various polymers are observed. The changes of polymer properties may be attributed to the increased crosslinking density. Other changes, such as to moisture resistance and hydrophobicity, are also observed [16,17,18].

However, not all of these materials can be used in medical radiation shielding constructions or individual radiation protection equipment, due to specific radiation protection requirements in the medical field [19]. It should also be noted that in many cases newly fabricated materials are hard solids, which are not flexible enough to secure conformal protection [20,21,22]. On the other hand, it is a challenge to produce homogeneous composite mixtures out of the component materials, especially if different concentrations of differently sized and shaped particles having different weights are embedded in a polymer matrix [23], since composite solidification takes some time and some heavy particles may settle to the bottom [24]. This problem may be resolved by applying a multi-layered shielding approach, e.g., using monolayers of metal particle/powder enriched polymers for the construction of a multi-layered structure [25]. In order to assess the X-ray attenuation properties of such layered polymer composites, and to further enhance the shielding ability of these structures, Monte Carlo simulations and genetic algorithms [26,27,28,29] can be used to design and fabricate a shielding material with a novel multilayered structure, wherein the characteristics of the multilayered structure should be fully exploited to ensure that the material exhibits an outstanding shielding ability against X-rays [30].

It should also be noted that the properties of the produced polymer composites largely depend on the technologies that can be used for the sample manufacturing process [31,32,33].

A performed literature analysis revealed that there is still a lack of information regarding the fabrication and application of advantageous multi-layered polymer composite constructions that are characterized by a corresponding X-ray attenuation and poses good mechanical and physical properties, enabling them to replace lead in radiation protection equipment and shielding constructions.

The aim of this work was to develop flexible lead-free multilayered polymer composites containing tin, cerium, tungsten, and bismuth compounds, and to characterize the X-ray attenuation and mechanical properties of these materials as possible candidates for the fabrication of medical radiation shielding equipment; separately and in a complex multilayered structure.

## 2. Materials and Methods

### 2.1. Fabrication of Experimental Samples

Experimental composites were prepared by mixing in equal parts by mass two components (A and B) of thermally-curable vinyl terminated polydimethylsiloxane (PDMS) silicone rubber, Endeavour T-1006 (Endeavour Enterprise Co., Taipei, Taiwan), and adding corresponding metal/metal oxide additives: Sn, powder size ~44 µm (Alfa Aesar, Heysham, UK); CeO_2_, powder size < 5 μm (Sigma Aldrich, Taufkirchen, Germany); WO_3_, powder size < 25 μm (Sigma Aldrich, Taufkirchen, Germany) and Bi, powder size ~44 µm (Alfa Aesar, Heysham, United Kingdom). The dynamic viscosity of the mixture of silicone components A and B was ~7000 cP. No additional solvent was used for adjusting the viscosity of the PDMS mixture. Due to the fact that the application of pure metals and metal oxides implicates some confusion in the estimation of the metal concentration in the composite and taking into account that the X-ray attenuation by oxygen is almost negligible, due to its low atomic number (Z = 8), it was decided to use the same number of moles for each metal component (0.002, 0.004, 0.008) and to calculate the molality concentration of metals in all four PDMS-based polymer composites, forming a multi-layered sample structure. Three different molality concentrations of metals were used for fabrication of monolayer composites. The composition of experimental monolayers by weight is provided in the Table 1.

**Figure 1:**
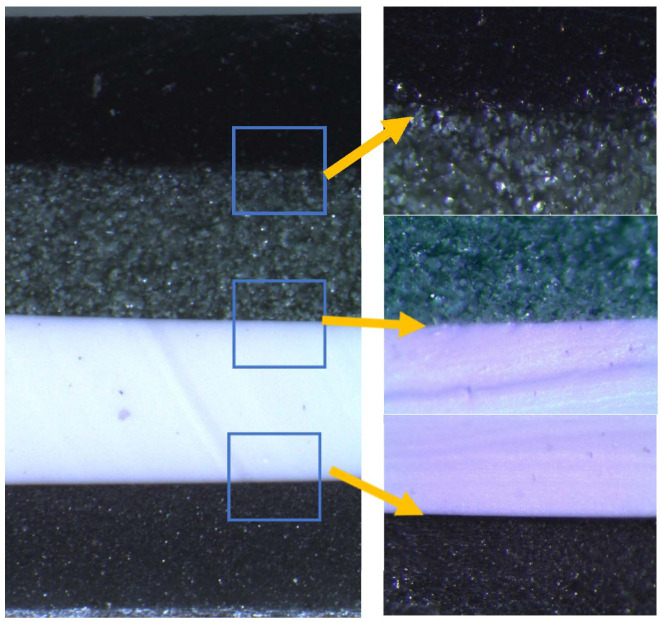
Cross-section of the multilayer composite and the interface between layers.

Polymer composites for each layer were mixed separately using a laboratory overhead stirrer LS-2000 (IRIS Analytical, Miami, FL, USA) equipped with a Teflon coated 25-mm diameter impeller (mixing speed−60 rpm, duration ≥3 min). Thoroughly mixed composites were poured into a special form (160 mm × 15 mm), which was constructed following the requirements of the ISO 6721 standard, in order to investigate the mechanical properties of layered constructions. The filled form was placed in a ultrasound bath Sono Swiss SW3H cleaner (ultrasonic frequency−38 kHz, effective ultrasonic power−80 W) for 360 s, for removal of air bubbles from the composite’s volume and from its inner surface, which contacted an inside bottom surface of the form. After this procedure, the form with composite was placed in a furnace for 10 min at 100 °C for curing and then cooled at room temperature outside the furnace. At such temperature, curing is accelerated, to avoid sedimentation of metal and metal oxide additives in the polymer matrix. For constructing a multilayer structure, next, thoroughly mixed composite was poured into the form on top of the already molded monolayer, following the same monolayer formation procedure as described above. The procedure was repeated until the total 4-layer construction was formed. Figure 1 shows the multilayer composite cross-section and interface between layers captured with an Olympus SZX7 microscope (Olympus Corporation, Tokyo, Japan). There are no visible inhomogeneities through the sample cross-section.

### 2.2. Material Characterization

**Figure 2:**
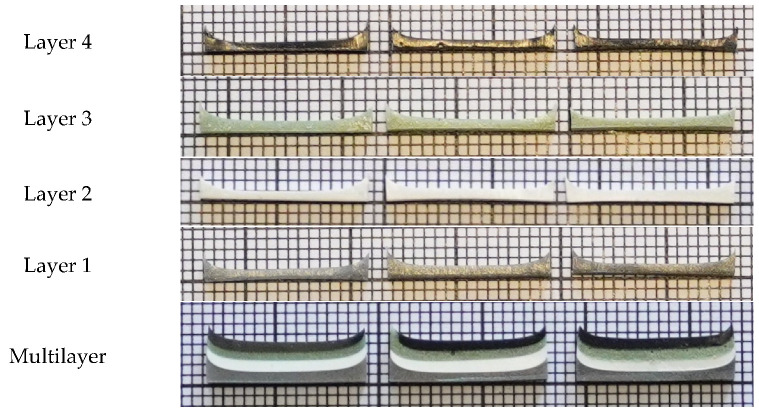
Experimental composites: Layer 1—PDMS + Sn; Layer 2—PDMS + CeO_2_; Layer 3—PDMS + WO_3_; Layer 4—PDMS + Bi; Multilayer structure—all layers starting with Layer 1 from the bottom side.

The thickness of each monolayer in the multilayer composite samples and of the multilayer structure itself were evaluated using ImageJ software. Millimeter scale was used for thickness evaluation. A photograph illustrating the thickness evaluation of the experimental polymer composites is provided in Figure 2, and the evaluated thicknesses of monolayers of different metal molality concentrations and multilayer composites are indicated in Table 2.

Since, one of the most important parameters used for the comparison of different radiation protection equipment, providing lead equivalency, is area density (the minimum mass per unit area of the protection material as defined in IEC 61331.3-2014), calculated area density values for experimental mono- and multilayer polymer composites are provided in Table 3, together with the mass density values of these composites.

Comparison of medical radiation protection garments with lead equivalency is based on the area density of the material, which enables quick assessment of the garment’s weight, which plays an important role for the staff involved in medical diagnostic examinations. Evaluation of the area density for each newly formulated polymer composite separately was carried out for comparison of their weight. A comparison was possible due to the fact that the fabricated layers of different composites having the same metal additive molality were relative thin and had approximately the same thickness. However, it should be noted that the thickness of the sample plays an important role, and the parameter defining area density of a multilayered material cannot be evaluated at all, because each layer has its own area density. Evaluation of physical density is a better choice for materials comparison under these circumstances. It was found that the average multilayer composite density (1.474 g/cm^3^) obtained at the highest molality concentrations of metal additives in the composites was lower compared with the density of lead-containing medical radiation protection aprons (2.13 g/cm^3^). However, the newly fabricated samples were almost twice as thick as medical aprons; thus, raising possible flexibility problems from wearing garments made out of these composites.

### 2.3. Analysis of Experimental Samples

#### 2.3.1. Evaluation of X-ray Attenuation Properties

The X-ray attenuation properties of the metal containing additives used for fabrication of polymer composites were simulated using the NIST XCOM database [34].

**Figure 3:**
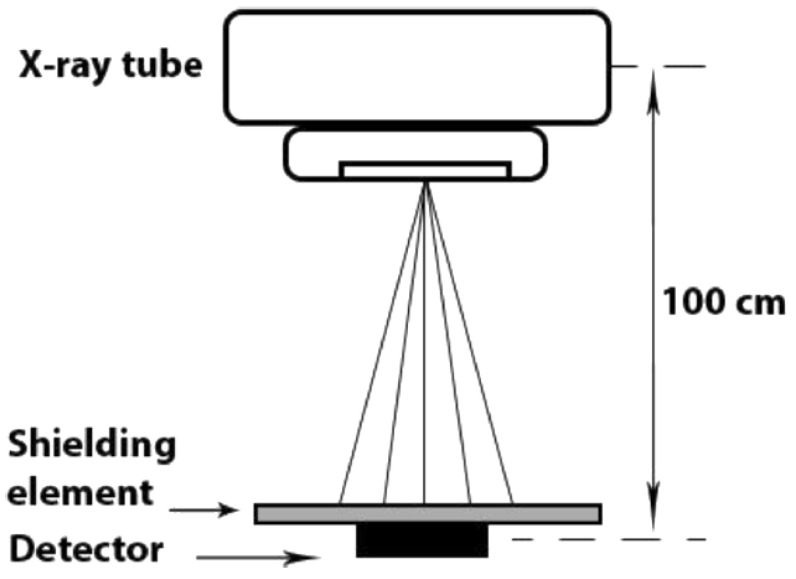
Experimental set-up for the evaluation of the X-ray attenuation properties of shielding elements.

X-ray attenuation of silicone composites containing Sn, CeO_2_, WO_3_, and Bi powders was experimentally investigated, following internationally accepted guidelines [35,36,37] and using the experimental set-up shown in Figure 3.

In order to assess the X-ray attenuation in experimental samples, these samples were placed in a direct irradiation field (100 mm × 100 mm) produced by a diagnostic X-ray machine AXIOM ICONOS R200 (Siemens Healthcare GmbH, Erlangen, Germany) on the top of a “Piranha” detector (RTI electronics, Mölndal, Sweden). A distance of 100 cm was set between the X-ray tube focal spot and the detector. Several X-ray tube voltages, covering the conventional X-ray diagnostic range (40, 60, 81, 100, 121, and 141 kV), were selected for X-ray attenuation analysis using the same exposure of 10 mAs.

In the first step, KERMA in air was measured directly irradiating the detector (experimental geometry without placement of the experimental samples); the dose beneath every experimental sample was measured in the second step (Figure 3).

X-ray transmission coefficient *B*(*x*) was evaluated using dose and KERMA ratio:

(1) $$B\left(x\right)=\frac{D\left(x\right)}{K\left(0\right)}\;$$

where *K*(0) is the air KERMA and *D*(*x*) is the measured dose beneath the shielding element (experimental sample). Dose measurements were performed in at least five locations beneath the samples.

#### 2.3.2. Tensile Tests of Experimental Samples

**Figure 4:**
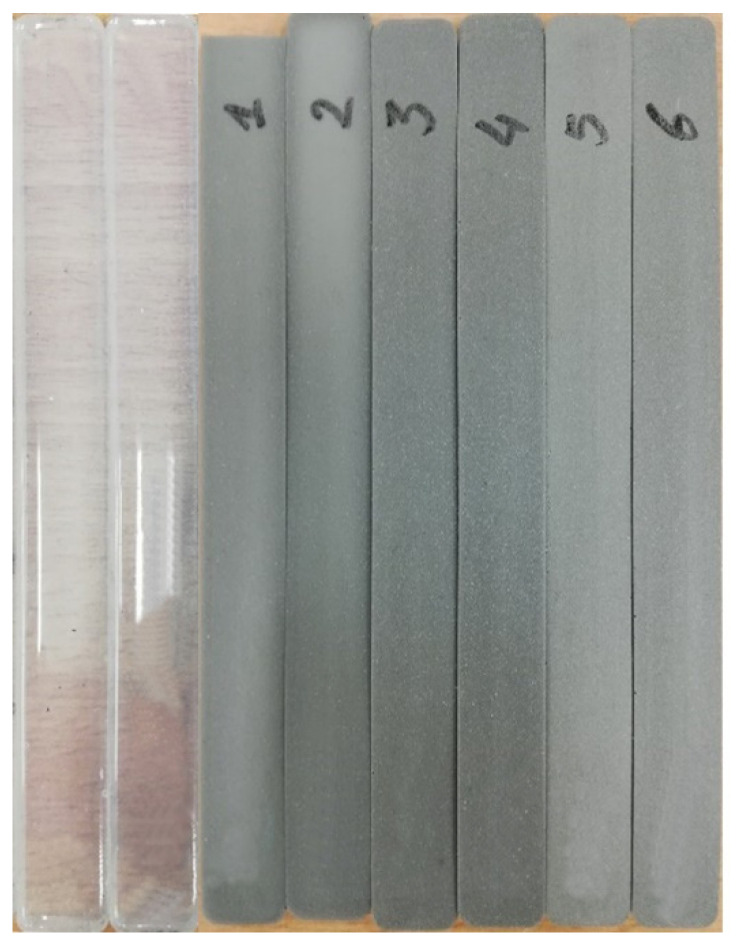
Experimental samples prepared for tensile tests, following ISO 6721 standard requirements. From the left: pure silicone samples; 1 and 2—0.8 mmol/g multilayer silicone composites samples; 3 and 4—1.6 mmol/g multilayer silicone composites samples; 5 and 6—3.2 mmol/g multilayer silicone composites samples.

Tensile tests (static and cyclic) for multilayer composites were conducted, in order to assess the mechanical properties of the experimental silicon- based multilayer composites, which were fabricated following the requirements of the ISO 6721 standard, as was described in Section 2.1. Each experimental sample was 160 mm long, 15 mm wide, and approximately 4.0 mm thick (Table 2). Tests with experimental composites were performed in an ElectroPuls E10000 Linear-Torsion (Instron, Norwood, MA, USA) machine, following ISO 6721 standard requirements. A photograph of the multilayer silicone composite samples produced using three different metal molalities (0.8, 1.6, and 3.2 mmol/g), and prepared for the tensile tests, is provided in Figure 4. For comparison, tensile tests were also performed with pure silicone samples, which were formed using the same amount of silicone as was used for fabrication of the multilayer composites. A maximum elongation of 50 mm was set for static tensile examinations, keeping a 50 mm/min elongation rate.

The cyclic tension (fatigue) examination was performed with experimentally evaluated elongation of tested samples. In order to achieve more homogeneous results, multilayer silicone composites were extended up to 30 mm, and an amplitude of ±20 mm was set. Cyclic tension examinations were repeated 40,000 times with a frequency of 1.0 Hz.

#### 2.3.3. Artificial Sweat Test

Artificial sweat testing of the experimental composites was performed, in order to assess possible release of nanoparticles from the polymer composites during some period of shielding material (silicone-based composite with metal additives) use. The test was performed and the multilayer composite samples were investigated following the regulations of the ISO105-E04-2008E standard. Monolayers of the highest molality concentration (3.2 mmol/g) were immersed in freshly prepared alkaline solution, using grade-3 water complying with ISO 3696 standard and containing 0.5 g of L-histidine monohydrochloride monohydrate (C_6_H_9_O_2_N_3_·HCl·H_2_O), 5 g of sodium chloride (NaCl), and 5 g of disodium hydrogen orthophosphate dodecahydrate (Na_2_HPO_4_·12H_2_O) per liter. Then, 0.1 mol/L of sodium hydroxide solution was added, achieving pH = 8 ± 0.2. The artificial sweat test was performed for durations of 24 h (1 day), 168 h (7 days), 336 h (14 days), and 744 h (31 day). Chemical analysis of samples was performed using inductively coupled plasma-mass spectrometry Optima 8000 (Perkin-Elmer, Waltham, MA, USA).

## 3. Results and Discussion

### 3.1. X-ray Attenuation Properties

**Figure 5:**
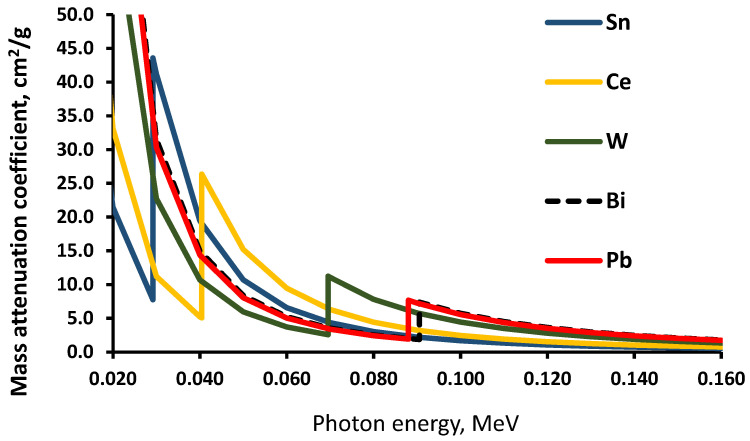
XCOM simulated total attenuation of photons in the different metals.

It is known [32] that elements with high Z (metal additives in polymer composites in our case) contribute significantly to the absorption of X-rays in the low energy region up to 100 keV, due to the existence of K edges in this energy interval. For this reason, the X-ray absorption/attenuation properties of composite metal additives Sn, Ce, W, and Bi were simulated using the XCOM database (Figure 5) and compared with the attenuation properties of Pb, which is still used in radiation protection/shielding equipment.

XCOM simulation results showed that, due to K-edges of the selected metals (29.2 keV for Sn, 40.4 keV for Ce, 69.5 keV for W, and 90.5 keV for Bi), all these metals showed a higher X-ray absorption ability than the pure lead (K-edge—88 keV) in certain energy intervals, thus giving an advantage for the proposed multilayer composite structures over lead or leaded rubber in terms of X-ray attenuation within the energy interval from 30 keV to 90 keV.

**Figure 6:**
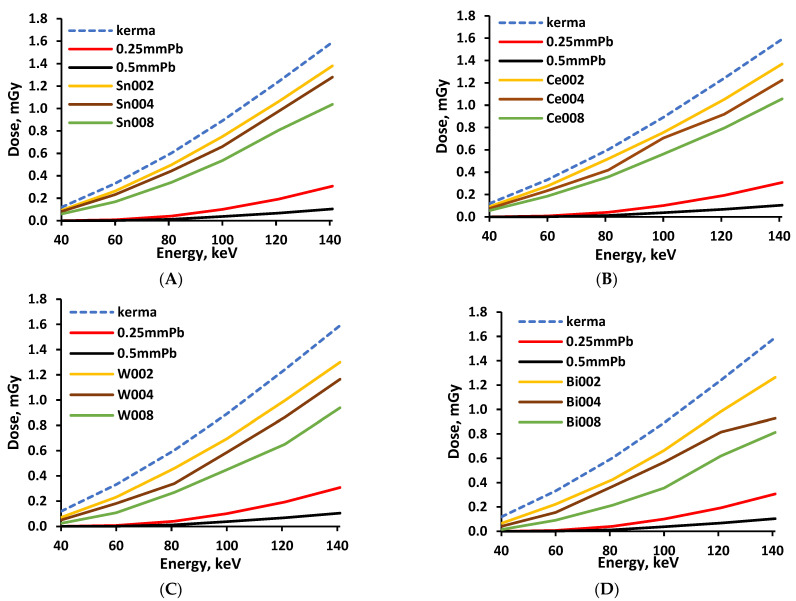
Doses measured beneath experimental samples: (**A**)—composite with Sn additives; (**B**)—composite with Ce additives; (**C**)—composite with W additives; (**D**)—composite with Bi additives. Numbers 002, 004, and 008 indicate the number of metal moles used for fabrication of the silicone composite.

Experimental evaluation of the X-ray attenuation properties of polymer composites was based on dose measurements performed without and with shielding material on the top of the detector, using the experimental set-up shown in Figure 3. In order to assess the effectiveness of X-ray attenuation, every polymer composite layer was evaluated separately, comparing the measured dose beneath the experimental composite, with the dose measured under same conditions beneath the lead-containing material, which provided 0.25 mmPb and 0.5 mmPb lead-equivalent radiation protection. Pieces of conventional medical radiation protection apron made out of leaded rubber were used as a lead-containing material. The results of the performed measurements for monolayer composites are provided in Figure 6A–D.

A similar increasing dose tendency was observed in every monolayer containing different metal additives when the X-ray energy was increased from 40 keV to 141 keV (medical diagnostic range). An increase of the filler amount in the polymer composite resulted in better X-ray attenuation properties. Lower dose values were measured beneath polymer composite’s monolayer containing higher Z additives, but these values were still higher compared with the doses obtained when measuring the 0.25 mmPb and 0.5 mmPb equivalent apron materials. The same tendencies were also observed for the multilayer composite samples. However, it is important to add that the multilayered samples were arranged starting with Layer 1 (composite containing metallic additives with the lowest Z) at the bottom, and finishing with Layer 4 (composite containing metallic additives with the highest Z) on the top. Sample exposure to X-rays was performed from the bottom side (Layer 1). The concept of multilayer sample formation and irradiation is based on the fact that low Z elements absorb low energy photons more efficiently compared with high Z elements and may significantly reduce the number of low energy waste photons that enter below the situated monolayers of a multilayer composite sample [32]. The discussed arrangement of layers fits well with the findings of McCaffrey et al. [21], who observed that low-Z materials upstream/high-Z materials downstream could yield up to five times more attenuation than that of the reverse order at 50 keV. It was also shown that such an arrangement was valid up to 150 keV.

**Figure 7:**
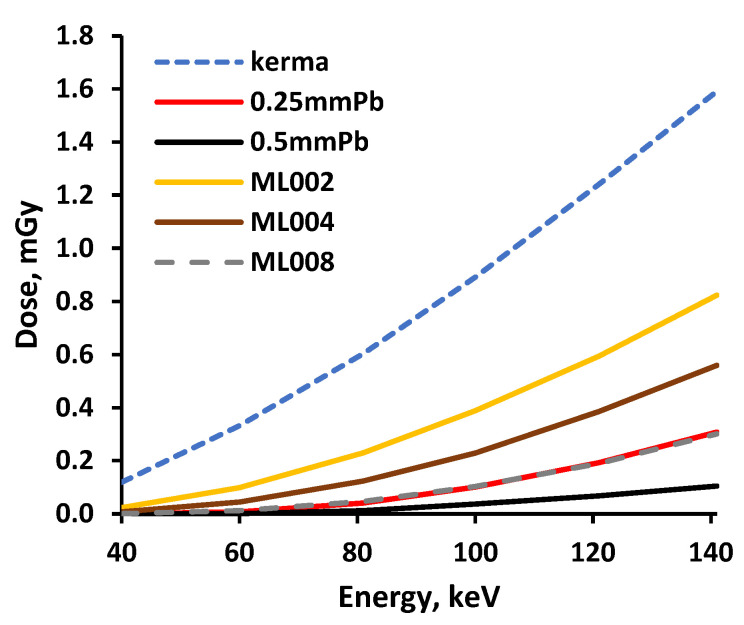
Doses measured beneath the experimental multilayer composites.

**Figure 8:**
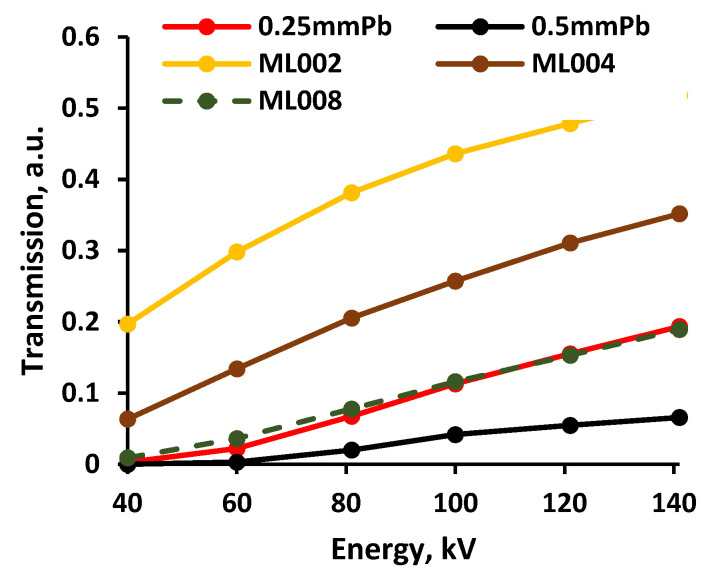
X-ray transmission coefficient variation in multilayer composites.

The total ionizing dose passing through the multilayered shielding construction (multilayer composite), measured beneath the multilayer composite, and the X-ray transmission coefficient’s variations within the energy range of interest are provided in Figure 7 and Figure 8, respectively.

The growth tendency of the experimentally evaluated doses with increasing energy was similar to those provided by the genetic algorithm for low dose and low energy interval [32]. Analysis of the attenuation properties of the multilayer composite samples revealed that the samples constructed from polymer composites containing the highest molality of metals (3.2 mmol/g) provided almost the same, or better, attenuation of X-rays within the energy range from 40 keV to 141 keV, compared with the leaded X-ray protective apron characterized by a 0.25 mmPb equivalency.

### 3.2. Results of Tension Examinations

**Figure 9:**
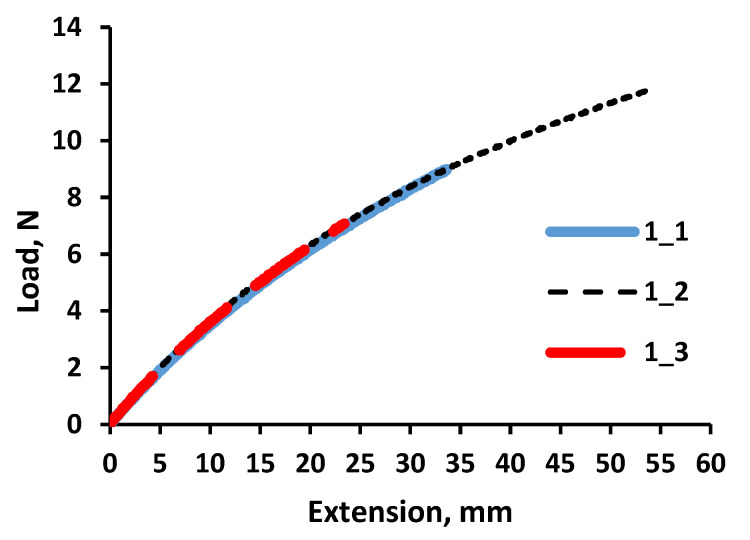
Tensile curves of pure silicone.

A static tension diagram, for further analysis of cyclic tension examinations, was evaluated to randomly test the pure silicone samples. Experiments were performed for randomly selected displacement (with a max displacement of 50 mm). There was no discontinuation of samples observed during the tests. The deformation curves of all three investigated silicone samples were also almost identical, independently of the test stopping moment, with the largest indicated elongation for sample 1_2 (Figure 9).

**Figure 10:**
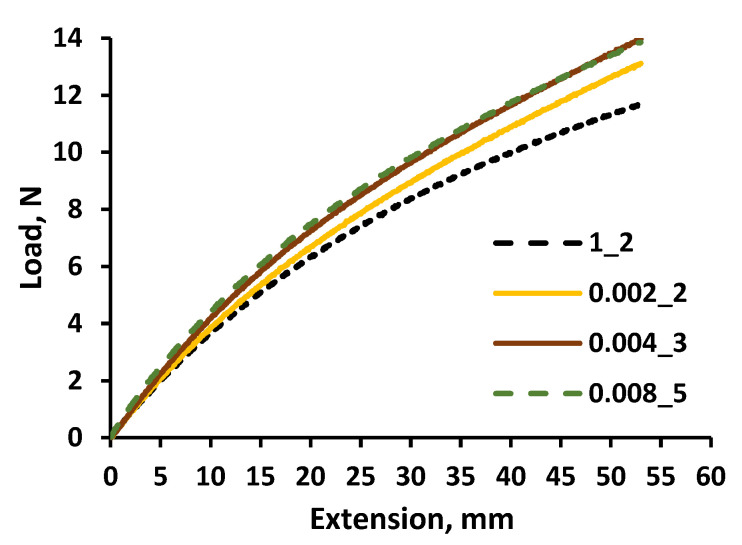
Tensile curves of different molality concentration multilayer silicone composites and pure silicone.

The same tests were performed with the multilayer silicone composites (Figure 10). It was found that the load required to achieve the same elongation of composites and the silicone samples was slightly increased with the increasing molality concentration of metallic additives in the polymer composites. A load difference of 2 N was indicated between pure silicone and the multilayer composite samples, with the highest metal molality concentration (3.2 mmol/g) at the maximum elongation.

**Figure 11:**
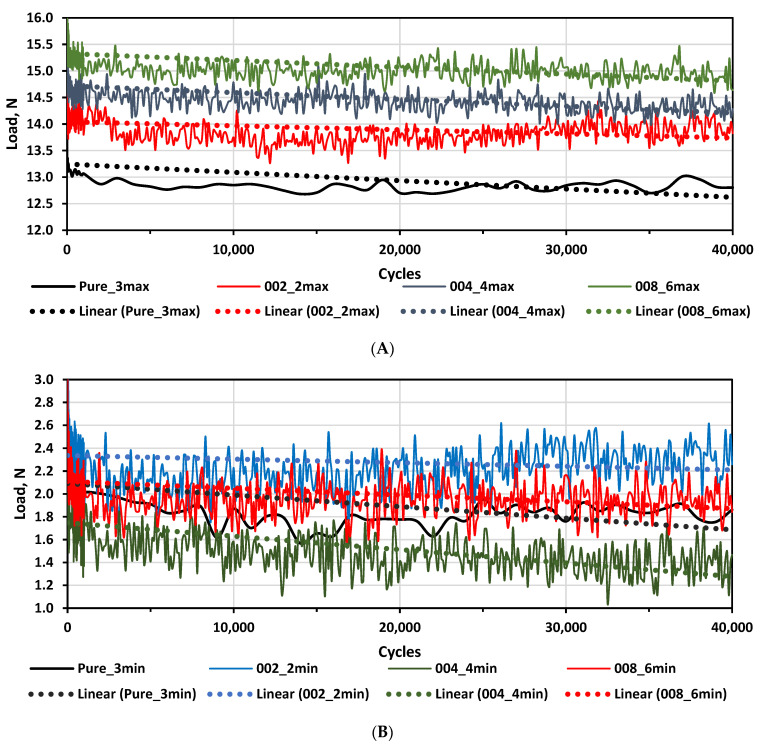
Loads vs. cycles for different multilayer composites and silicone: (**A**)—max load vs. cycles; (**B**)—minimum load vs. cycles.

The cyclic tension (fatigue) was controlled by the maximum elongation. Variations of minimum and maximum load over the cyclic examinations of multilayer composites and pure silicone samples are shown in Figure 11.

Since the multilayer composite samples structure, in general, was not stiff, the load power needed to achieve the same elongation changed over the cycles. Taking this into account, trendlines were used for the evaluation of load power changes over the cycles. It was found that the load power required to reach a pre-set max elongation during the tests was slightly increased with the increasing metal molality in experimental composites; however, a slightly decreased overall tendency of power load was observed over all test cycles, indicating some very small deteriorations of the sample’s mechanical properties. There was no clear relationship identified between the load power needed to achieve minimum elongation and the metal molality of the polymer composites, but a similar decreasing tendency of power load was observed in all test cycles.

### 3.3. Results of Artificial Sweat Tests

**Figure 12:**
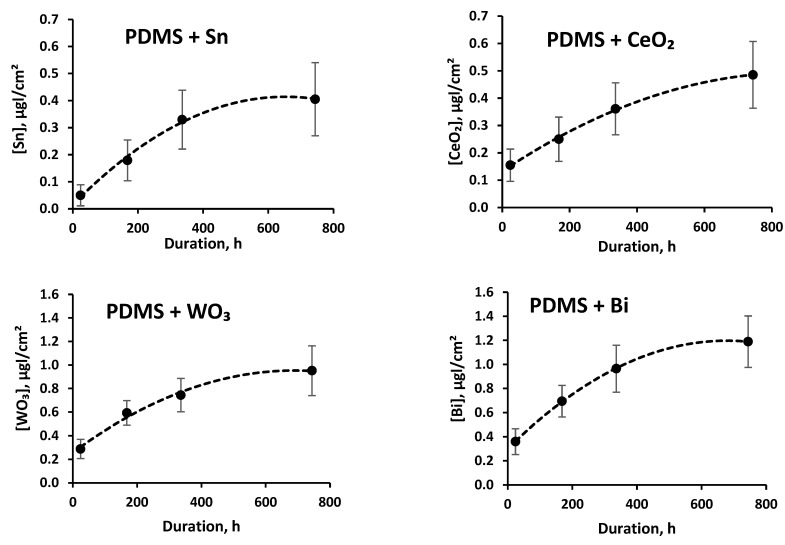
Filler amount per unit of surface area released from the polymer matrix during artificial sweat tests.

The released amounts of tin, cerium oxide, tungsten oxide, and bismuth from the silicone polymer matrix in artificial sweat solution after sample immersion for a time period of up to one month are presented in Figure 12.

It was found that the release of fillers from the polymer matrix immersed in artificial sweat solution was slightly increased over the time. After 31 days testing, the highest release of 1.189 ± 0.213 µg/cm^2^ was found for bismuth. The released amount of bismuth was rather low, since it amounted to only 0.0017% of the Bi material that was used for fabrication of the experimental composite samples. The lowest release of 0.485 ± 0.122 µg/cm^2^ was observed for tin.

## 4. Conclusions

New lead-free multilayer polymer composites for radiation shielding, consisting of two parts (A and B) thermally curable vinyl-terminated polydimethylsiloxane silicone rubber and tin, cerium oxide, tungsten oxide, and bismuth powders, were fabricated, varying the filler molality concentration in the polymer matrix.

Preliminary XCOM simulations showed that the fabrication of the multilayer silicone composites using thoroughly selected metallic fillers for each layer has an advantage over lead in terms of X-ray attenuation in the diagnostic energy range, due to the existing K-absorption edges of the selected metals (29.2 keV for Sn, 40.4 keV for Ce, 69.5 keV for W, and 90.5 keV for Bi). All these metals indicated a higher X-ray absorption ability than pure lead (K-absorption edge—88 keV) in certain energy intervals, covering the whole medical diagnostic range.

The performed experimental investigation revealed that the multilayer composites fabricated from separate silicone composite layers, containing the highest molality concentration of 3.2 mmol/g of different metallic fillers demonstrated almost the same or even better X-ray attenuation properties as medical radiation protection aprons characterized by 0.25 mmPb equivalency, in the diagnostic energy range between 40 keV and 141 keV.

The performed static and cyclic tension tests of multilayer polymer composites have shown that a higher power load should be applied for composites containing higher molality concentration of metallic fillers, if the same deformation is to be achieved. In addition, the deterioration tendency of the composite mechanical properties was observed after 40,000 deformation cycles.

The artificial sweat test revealed that the amount of metallic additives released from the polymer matrix over a one month period was very low; thus, indicating that there will not be any impact on the X-ray attenuation properties of the newly fabricated shielding composites over a long period of time.

The performed investigation of X-ray attenuating and mechanical properties of the newly developed lead-free multilayer polymer composites revealed the potential of these composites in the application of these materials as toxic lead substitutes for the construction of shielding elements against ionizing radiation. Polymer mass reduction, keeping the same amount of metallic additives in the composite, may be an elegant solution for the development of thinner multilayer polymer composites with the required X-ray attenuation properties; however, in this case, some deterioration of mechanical properties will be inevitable.

## Figures and Tables

**Table 1:** The composition of experimental monolayers by weight.

Metal Molality, mmol/g	Number of Metal Moles	Monolayer Weight, g			
		Layer 1 PDMS + Sn	Layer 2 PDMS + CeO_2_	Layer 3 PDMS + WO_3_	Layer 4 PDMS + WO_3_
0.8	0.002	2.5 + 0.236	2.5 + 0.344	2.5 + 0.464	2.5 + 0.418
1.6	0.004	2.5 + 0.473	2.5 + 0.688	2.5 + 0.927	2.5 + 0.836
3.2	0.008	2.5 + 0.945	2.5 + 1.377	2.5 + 1.855	2.5 + 1.672

**Table 2:** Thickness of the prepared polymer composites.

Molality, mmol/g	Thickness, mm				
	Layer 1 PDMS + Sn	Layer 2 PDMS + CeO_2_	Layer 3 PDMS + WO_3_	Layer 4 PDMS + Bi	Multilayer
0.8	0.695	0.912	0.730	0.731	3.949
1.6	0.888	1.033	0.801	0.873	4.031
3.2	0.919	1.072	0.847	1.006	4.161

**Table 3:** Physical density and area density of experimental composites.

Polymer Composite	Molality, mmol/g	Density, g/cm^3^	Area Density, g/cm^2^
PDMS + Sn	0.8	1.082	0.114
	1.6	1.158	0.124
	3.2	1.312	0.144
PDMS + CeO_2_	0.8	1.115	0.118
	1.6	1.228	0.133
	3.2	1.440	0.162
PDMS + WO_3_	0.8	1.154	0.123
	1.6	1.304	0.143
	3.2	1.577	0.181
PDMS + Bi	0.8	1.148	0.122
	1.6	1.292	0.139
	3.2	1.562	0.174
Multilayer	0.8	1.125	-
	1.6	1.246	-
	3.2	1.474	-

## Data Availability

The data in this work are available upon request from the corresponding author.
